# Mesenchymal Stromal Cells Suppress T-Cell-Mediated Delayed-Type Hypersensitivity via ALCAM-CD6 Interaction

**DOI:** 10.1093/stcltm/szad012

**Published:** 2023-04-17

**Authors:** WonKyung J Cho, Sharad K Mittal, Sunil K Chauhan

**Affiliations:** Schepens Eye Research Institute of Mass Eye and Ear, Harvard Medical School, Boston, MA, USA; Schepens Eye Research Institute of Mass Eye and Ear, Harvard Medical School, Boston, MA, USA; Schepens Eye Research Institute of Mass Eye and Ear, Harvard Medical School, Boston, MA, USA

**Keywords:** mesenchymal stromal cells, immunomodulation, T cells, alloimmunity

## Abstract

Mounting evidence suggests mesenchymal stromal cells (MSCs) suppress CD4^+^ T-cell activation, but whether MSCs directly regulate activation and expansion of allogeneic T cells has not been fully deciphered. Here, we identified that both human and murine MSCs constitutively express ALCAM, a cognate ligand for CD6 receptors on T cells, and investigated its immunomodulatory function using in vivo and in vitro experiments. Our controlled coculture assays demonstrated that ALCAM-CD6 pathway is critical for MSCs to exert its suppressive function on early CD4^+^CD25^−^ T-cell activation. Moreover, neutralizing ALCAM or CD6 results in the abrogation of MSC-mediated suppression of T-cell expansion. Using a murine model of delayed-type hypersensitivity response to alloantigen, we show that ALCAM-silenced MSCs lose the capacity to suppress the generation of alloreactive IFNγ-secreting T cells. Consequently, MSCs, following ALCAM knockdown, failed to prevent allosensitization and alloreactive T-cell-mediated tissue damage.

## Graphical Abstract

**Graphical Abstract:**
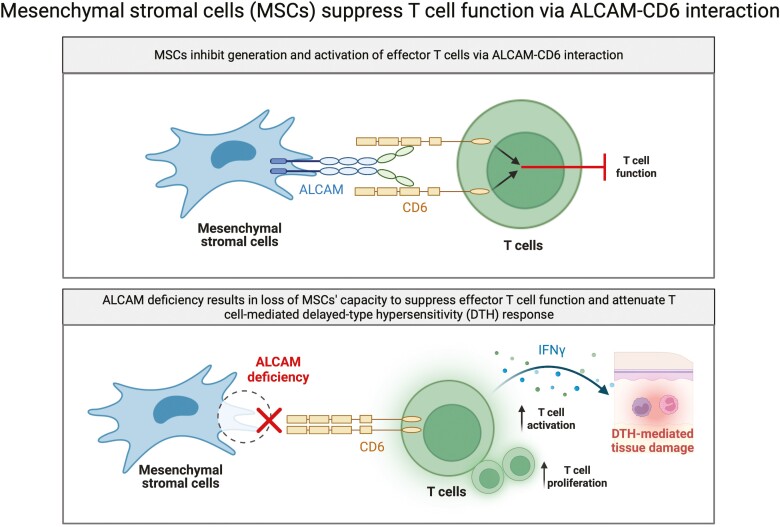

**Significance Statement**

The current study demonstrates, for the first time, that mesenchymal stromal cells exert their immunosuppressive function on allogeneic T cells via ALCAM. Silencing of ALCAM on mesenchymal stromal cells resulted in the loss of their capacity to suppress the generation of alloreactive effector T cells and attenuate T-cell-mediated delayed-type hypersensitivity response. The delineation of the mechanism by which mesenchymal stromal cells exert their immunoregulatory function provides novel insights into the development of MSC-based therapy to regulate alloimmunity.

## Introduction

Mesenchymal stromal cells (MSCs) are a heterogenous subset of non-hematopoietic progenitor cells capable of differentiating into cells of multiple lineages such as chondrocytes, myoblasts, and adipocytes.^1^ Since their first characterization, MSCs have largely been studied for their stem cell-like properties for utilization in tissue repair and regeneration. In recent years, however, MSCs have attracted attention for their immunomodulatory properties.^2^ MSCs dampen the inflammatory response, mediating both innate and adaptive immunity via regulating the effector function of various immune cells, including neutrophils, monocytes/macrophages, and dendritic cells.^3-5^ In a recent study, we have demonstrated that exogenous administration of MSCs suppresses immune-mediated graft rejection by directly interacting with T regulatory cells.^6^ Despite the advancements in the understanding of the immunomodulatory functions of MSCs, whether MSCs directly regulate the activation and expansion of allogeneic T cells have yet to be investigated.

Recent reports have suggested activated leukocyte cell adhesion molecule (ALCAM, CD166) as a phenotypic marker for human MSCs, but their functional contribution has not been studied.^7^ ALCAM is a glycoprotein belonging to the immunoglobulin superfamily and is the physiological ligand for CD6, a regulatory molecule primarily expressed by CD4^+^ T cells.^8,9^ The function of CD6 in regulating T cells has been a matter of debate, given the previous reports of CD6 not only promoting immune synapse formation but also suppressing early T-cell activation.^10-13^ Thus, we sought to determine the function of MSC-expressed ALCAM and its interaction with CD6 in mediating CD4^+^ T-cell response. We and others have previously shown that MSCs inhibit CD4^+^ Th1 response by suppressing APC maturation,^3,14^ but in this study, we investigated whether MSCs directly interact with T cells via ALCAM-CD6 pathway and suppress the activation and proliferation of alloreactive CD4^+^ T cells using a murine model of delayed-type hypersensitivity (DTH) response.

Our data demonstrate that both human and murine MSCs express ALCAM, via which MSCs interact with T cells to suppress their function and proliferation. Blockade of ALCAM-CD6 interaction using anti-ALCAM and anti-CD6 neutralizing antibody and selective gene silencing of ALCAM on MSCs resulted in abrogation of MSCs’ capacity to suppress CD4^+^ T cell response. Using both in vitro coculture assays and in vivo delayed-type hypersensitivity models, our study demonstrates that MSCs exert suppressive effects on alloreactive CD4^+^ T cells via ALCAM-CD6 interaction.

## Materials and Methods

### Animals

Six-to-8-week-old C57BL/6 and BALB/c mice (Charles River Laboratories, Wilmington, MA, USA) were used in the experiments. Mice were housed in a pathogen-free environment at the animal facility of Schepens Eye Research Institute. The protocol was approved by the IACUC, and all animals were treated in compliance with ARVO’s Statement for the Use of Animals in Ophthalmic and Vision Research.

### Delayed-Type Hypersensitivity Assay

Delayed type hypersensitivity assay was performed as reported previously.^15^ Briefly, to induce allosensitization, allogeneic BALB/c splenocytes (5 × 10^6^ splenocytes) suspended in normal saline were subcutaneously injected into the nape of the neck. C57BL/6 mice were injected intravenously with Mock- or ALCAM-silenced MSCs (C57BL/6) on days 0 and 1 post-sensitization. To assess the generation of alloreactive Th1 generation, draining lymph nodes were harvested on day 7 for ELISPOT analysis. In another set of the experiment to measure ear-swelling, the mice were re-challenged, on day 14 post-sensitization, by epicutaneous injection of mitomycin-C treated BALB/c splenocytes (4 × 10^6^ splenocytes) suspended in normal saline to the right ear. The left ear was injected with saline alone to serve as intra-mouse control. Ear thickness was measured pre-sensitization and at 24 and 48 h post-challenge using a dial thickness gauge (Mitutoyo, Kawasaki, Japan). The degree of swelling was calculated as the difference in thickness of the challenged ear (right ear) minus the baseline line thickness of the unchallenged ear (left ear). The mice were sacrificed 48 h post-challenge and the ear and draining lymph nodes were harvested for H&E and flow cytometry analysis.

### Isolation, Expansion, and Characterization of MSCs

Using the plastic adherence method, MSCs were purified and expanded from bone marrow cells harvested from femurs of C57BL/6 mice in murine MesenCult medium with supplements (STEMCELL Technologies, Canada).^4,16^ Non-adherent cells were removed every 48 h by changing the medium. Phenotypic and functional characterizations of MSCs, as set forth by “The International Society for Cellular Therapy,” were completed prior to use.^17^ Human bone marrow-derived MSCs (STEMCELL Technologies) were purchased and expanded using MesenCult™-ACF Plus Culture Kit (STEMCELL Technologies). MSCs from the second or third passage were used in the following experiments.

### Short hairpin RNA Gene Silencing

Murine MSCs (1.5 × 10^6^) were seeded in a 75-cm^2^ flask to achieve 60%–70% confluency. The cells were transfected with ALCAM-specific or control short hairpin RNA (shRNA) as per the manufacturer's protocol (Santa Cruz Biotechnology, TX, USA). After overnight incubation, transfection media was removed and MSCs expressing stable shRNA clones were selected by culturing in MSC media supplemented with puromycin (5 μg/mL) for an additional 72 h. Gene silencing was confirmed by performing real-time PCR (data not shown) and flow cytometry analysis post-transfection (Fig. 4A).

### Intravenous Administration of MSCs

In vitro expanded and characterized MSCs (0.5 × 10^6^ cells suspended in 100 μL sterile saline), either treated with control shRNA or ALCAM shRNA (Santa Cruz Biotechnology), were injected intravenously via tail vein on days 0 and 1 post-priming.^18^

### Single-Cell Suspensions

Single-cell suspension of tissues harvested from ears was prepared using a published protocol.^19^ In brief, the inflamed area of the ear was cut using a 3 mm trephine, and the dorsal and ventral side of the ear pinnae were separated to gently scape of the cartilage. The tissues were then digested in incomplete RPMI (Lonza, MD, USA) containing 1% FBS, collagenase type IV (300 μg/mL; Sigma–Aldrich, MO, USA), and DNase I (50 U/mL; Roche, Switzerland) for 90 min at 37 °C. Digested tissues or lymph nodes were filtered through a 70‐μm cell strainer to make single-cell suspensions. Draining cervical lymph nodes were harvested on day 7 following alloantigen priming and filtered through 70‐μm cell strainer to make single-cell suspensions, as reported previously. Single-cell suspensions were analyzed using flow cytometry and ELISPOT, as described below.

### Flow Cytometry Analysis

**Figure 1.**
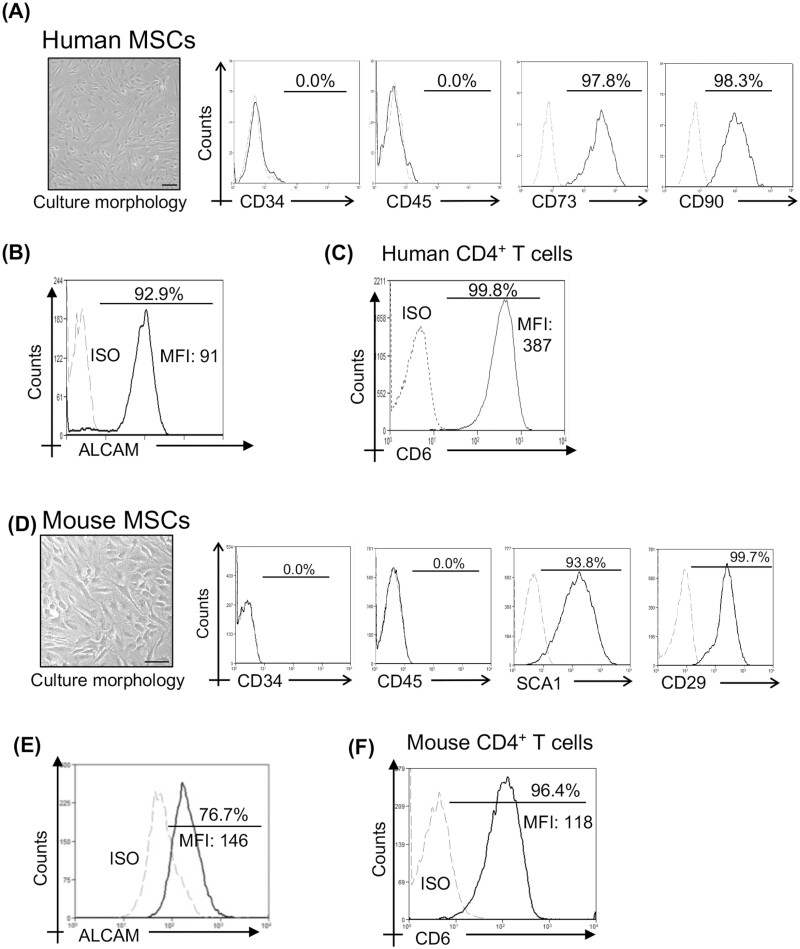
Constitutive expression of ALCAM on human and murine MSCs and its receptor CD6 on CD4^+^CD25^−^ T cells. (**A**) Representative bright-field image (left) showing the morphology of bone marrow-derived human MSCs (scale bar = 100 μm). Flow cytometry histograms (right) depicting negative expression of hematopoietic cell marker (CD45 and CD34) and positive expression of stem cell marker (CD73 and CD90) on human MSCs at the third passage. Dotted line, isotype control; solid line, antibody staining. (**B**) Flow cytometry histogram showing ALCAM expression on human MSCs. (**C**) Flow cytometry histogram showing CD6 expression on CD4^+^CD25^−^ T cells purified from human peripheral blood mononuclear cells. (**D**) Representative bright field image showing the morphology of human MSCs (left; scale bar = 100 μm) and flow cytometry plots (right) depicting negative expression of hematopoietic cell marker (CD45 and CD34) and positive expression of stem cell marker (SCA1 and CD29) on bone marrow-derived mouse MSCs at second passage. (**E**) Histogram showing ALCAM expression on mouse MSCs. (**F**) Flow cytometry histogram showing CD6 expression on CD4^+^CD25^−^ T cells isolated from mouse splenocytes.

**Figure 2.**
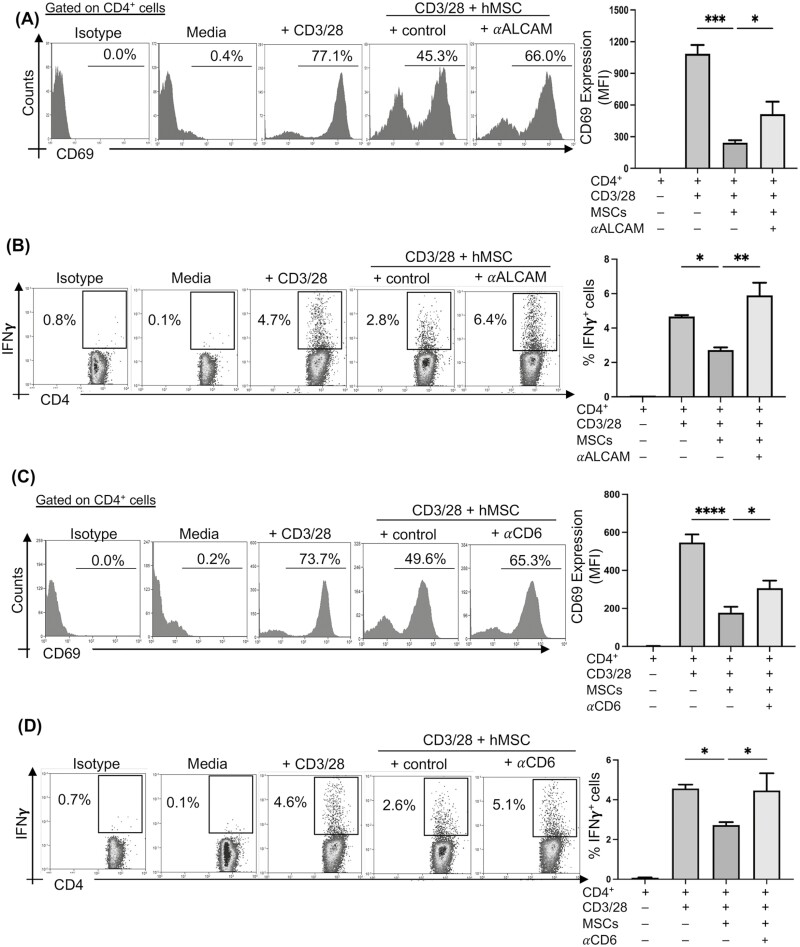
ALCAM or CD6 neutralization results in the abrogation of MSC-mediated suppression of T-cell activation CD4^+^CD25^−^ T cells were magnetically sorted from human peripheral blood mononuclear cells and stimulated with anti-CD3/CD28-coated beads at a 1:1 ratio and cultured on the monolayer of human MSCs (hMSCs). hMSCs were pre-treated with or without ALCAM-neutralizing antibodies. (**A**) Representative flow cytometry histogram (left) and bar chart (right) demonstrating expression of CD69 (median fluorescence intensity; MFI) by CD4^+^ T cells in the indicated groups of cultures at 24 h. (**B**) Representative flow cytometry dot plots (left) and cumulative bar chart (right) showing frequencies of CD4^+^IFNγ^+^ Th1 cells (gated on CD4^+^ cells) in the indicated groups of cultures at 48 h of coculture. In another set of experiments, CD4^+^CD25^−^ T cells were pre-treated with CD6 neutralizing antibody or control isotype prior to coculture. (C) Representative flow cytometry histogram (left) and bar chart (right) demonstrating expression of CD69 (MFI) by CD4^+^ T cells in the indicated groups of cultures at 24 h. (**D**) Representative flow cytometry dot plots (left) and cumulative bar chart (right) showing frequencies of CD4^+^IFNγ^+^ Th1 cells (gated on CD4^+^ cells) in the indicated groups of cultures at 48 h of coculture. Data from 3 independent experiments are shown. Data presented are mean ± SD. One-way ANOVA, *P < .05, **P < .01, ***P < .001, ****P < .0001.

Surface staining of single-cell suspensions was performed using fluorochrome-conjugated antibodies to characterize (1) CD45, CD34, CD73, and CD90 expression by human MSCs (Fig. 1A), (2) CD45, CD34, CD29, and SCA-1 expression by mouse MSCs (Fig. 1D) (3) CD4 and CD25 expression by T cells.^20,21^ Fluorochrome-conjugated CD69 antibody was used to assess early T-cell activation. To evaluate the intracellular expression of IFNγ in CD4^+^CD25^−^ Th1 cells, Brefeldin A (0.1 µL/100 µL media; Biolegend, CA, USA) was added to the indicated coculture systems 12 h prior to flow cytometry analysis. Stained cells were acquired using an LSR II flow cytometer (BD Biosciences, CA, ISA) and analyzed using Summit software (Dako Colorado Inc., Fort Collins, USA).

### In Vitro Cultures

For our in vitro experiment, T-cell (CD4^+^CD25^−^) population was magnetically sorted to exclude the regulatory T-cell population from single-cell suspensions of splenocytes of C57BL/6 mice or human peripheral blood mononuclear cells (PBMCs; STEMCELL Technologies) using CD4^+^CD25^+^ T-cell isolation kits (Miltenyi Biotech, CA, USA). The purity of the sorted cells (>95%) was confirmed by flow cytometry analysis prior to the coculture setup. T cells cultured alone served as a negative control. For coculture assays, ^-^ CD4^+^CD25^-^ T cells, stimulated with anti-CD3/CD28-coated Dynabeads (Thermo Fisher) at 1:1 ratio, were cultured on a monolayer of MSCs in the presence or absence of human anti-ALCAM neutralizing antibody (20 µg/mL; R&D Systems, CA, USA) for 24 and 48 h to assess early T-cell activation and Th1 generation, respectively. In a separate set of experiments, cocultures were treated with human anti-CD6 or isotype controls (20 μg/m; R&D Systems) for 24 and 48 h. For murine cocultures, stimulated T cells were cultured alone or on a monolayer of Mock or ALCAM-silenced MSCs.

### In Vitro Proliferation

To assess proliferation, sorted T cells, labeled with CellTrace CFSE proliferation kit (Thermo Fisher Scientific, MA, USA), were stimulated with anti-CD3/CD28-coated Dynabeads (Thermo Fisher) at 1:1 ratio and cultured on a monolayer of MSCs, as described above, for 72 h. T cells, harvested and surface stained for CD4 surface marker, were acquired using an LSR II flow cytometer (BD Biosciences). The proliferation index was calculated as total number of divisions divided by the number of cells that underwent division using FlowJo software (BD Biosciences).

### ELISPOT Assay

ELISPOT assay was used to measure the frequencies of directly primed IFNγ-producing T cells, as previously described.^22^ Briefly, a 96-well murine IFNγ ELISPOT plate was set up as per the manufacturer’s protocol (Immunospot, OH, USA). T cells were magnetically sorted from single-cell suspensions of draining lymph nodes harvested on day 7 from naïve or primed mice (C57BL/6) using the Pan T-cell isolation kit (Miltenyi Biotech). To quantify the frequencies of directly allosensitized T cells, T cells (C57BL/6) were incubated in triplicates with BALB/c splenic donor-derived antigen-presenting cells at 1:1 ratio. Antigen-presenting cells (APCs) were magnetically sorted from the single-cell suspension of splenocytes using CD90.2 isolation kit (Miltenyi Biotech). After 48 h of coculture, plates were washed and incubated with a biotinylated anti-IFNγ detection antibody. Spots were developed using the Blue developer solution (ImmunoSpot). The number of spots was counted and analyzed using the CTL ImmunoSpot S6 Entry M2 Analyze scanner and ImmunoSpot software (ImmunoSpot).

## Histological Analysis

Cross-sections were prepared from formalin-fixed ear pinnae harvested on day 2 post-rechallenge (day 16 post-priming) and stained with H&E. Pinnae tissue structure was analyzed under a brightfield microscope (Leica DM750). Pinnae thickness was measured from the epithelium to the cartilage (chondrocytes) using NIH ImageJ (version 1.34s) software.^23^

## Statistical Analysis

Unpaired 2‐tailed Student’s *t*-tests were used to compare means between 2 groups and Kruskal-Wallis one-way ANOVA tests were used to compare means between 3 or more groups. Significance was set at *P* < .05. Results are presented as mean of at least 3 independent experiments.

## Results

### Human and Murine MSCs Constitutively Express ALCAM, A Cognate Ligand of T-Cell CD6 Receptor

Human MSC characterization was confirmed by visualizing spindle shape morphology using phase-contrast microscopy and by evaluating for their positive expression of CD73 and CD90 (MSC markers) and negative expression of CD45 and CD34 (hematopoietic cell markers) using flow cytometry (Fig. 1A). Recent reports have found ALCAM expression to be a specific phenotypic marker of humans MSCs (hMSCs).^7^ Similar to previous reports, we observed constitutively high expression of ALCAM by human MSCs (Fig. 1B). Given that ALCAM exerts its effect by binding to its cognate receptor CD6 we assessed the expression of CD6 on peripheral CD4^+^CD25^−^ T cells. Flow cytometry analysis showed the majority of CD4^+^CD25^−−^ T cells expressed CD6 (Fig. 1C). Given our observation of constitutive expression of ALCAM on human MSCs, we next determined ALCAM expression in murine MSCs (mMSCs), which has yet to be investigated. mMSCs, purified and expanded from the bone marrow, were characterized for CD29^+^ SCA-1^+^ expression and CD45^−^ and CD34^−^ expression (Fig. 1D). Our data showed that mMSCs, akin to hMSCs, express substantial levels of ALCAM (Fig. 1E). Expression of their cognate receptor CD6 was confirmed in naïve CD4^+^CD25^−^ T cells isolated from murine splenocytes (Fig. 1F). Our data confirm the constitutive expression of ALCAM on hMSCs and demonstrate, for the first time, substantial levels of ALCAM on murine MSCs.

### Blockade of ALCAM-CD6 Interaction Inhibits MSC-Mediated Suppression of T-cell Activation

Despite ALCAM being suggested as a phenotypic marker of human MSCs, its biological function on MSCs in the context of alloimmunity has not been elucidated. Here, we investigated the effect of ALCAM-CD6 pathway on MSC–T-cell-interaction. CD4^+^CD25^−^ naïve T cells purified from human peripheral blood mononuclear cells by magnetic sorting were stimulated with anti-CD3/28 for 24 h on an MSC monolayer. Early T-cell activation marker CD69 was assessed on CD4^+^ cells using flow cytometry analysis (Supplementary Fig. S1). Our data showed that MSCs significantly suppressed the early activation of T cells (Fig. 2A). To determine the function of MSC-expressed ALCAM, we cocultured MSCs and CD4^+^CD25^−^ T cells in the presence or absence of ALCAM-neutralizing antibody. Flow cytometry analysis demonstrated that blockade of ALCAM resulted in the reversal of MSC-mediated suppression of T-cell activation (Fig. 2A). To assess whether MSCs modulate T effector function via MSCs, we next assessed the frequencies of IFNγ^+^ cells following 48 h of MSCs-T cells coculture in the presence of ALCAM-neutralizing antibodies. Our data demonstrated that MSCs significantly suppressed the frequencies of CD4^+^IFNγ^+^ cells, which was abrogated when ALCAM was neutralized (Fig. 2B). ALCAM-neutralizing antibody had no effect on CD3/28-mediated T-cell activation when added to CD4^+^ T cells cultured alone (Supplementary Fig. S2). Having observed that neutralization of ALCAM expression hinders MSC-mediated suppression of T-cell activation, we next investigated whether similar effects will be observed when the T-cell arm of the ALCAM-CD6 interaction is blocked. We performed similar cocultures of MSCs and CD4^+^CD25^−^ T cells, but in the presence of CD6-neutralizing antibodies or control isotypes for 24 and 48 h. Flow cytometry analyses demonstrated that neutralization of CD6 significantly reversed the MSC-mediated suppression of early T-cell activation (Fig. 2C) and T-effector function (Fig. 2D), compared to control cocultures. Taken together, our data demonstrate that ALCAM-CD6 interaction is critical to MSC-mediated suppression of T-cell activation.

### ALCAM-CD6 Neutralization Abrogates MSC-mediated Suppression of T-Cell Proliferation

**Figure 3.**
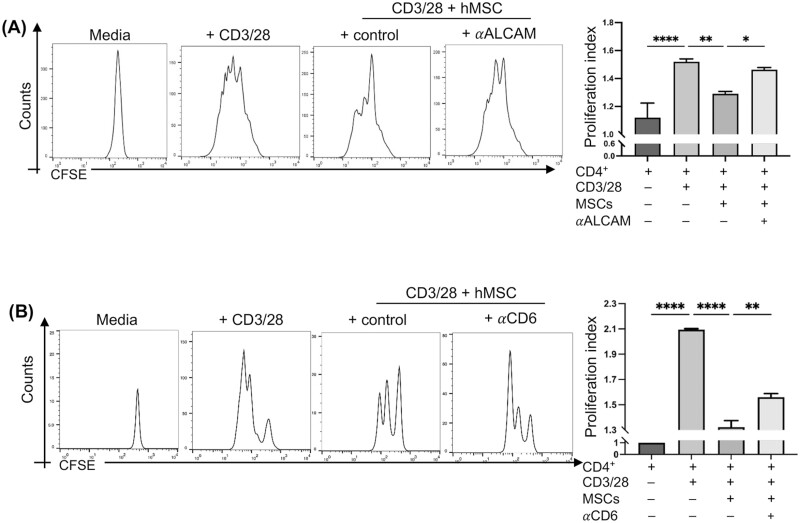
Blockade of ALCAM-CD6 interaction inhibits MSC-mediated suppression of T-cell proliferation CD4^+^CD25^−^ T cells were magnetically sorted from human peripheral blood mononuclear cells and labeled with CellTrace CFSE prior to the coculture assays. CFSE-labeled CD4^+^CD25^−^ T cells were stimulated with anti-CD3/CD28-coated beads at a 1:1 ratio on a monolayer of human MSCs (hMSCs) for 72 h. (**A**) hMSCs were treated with or without ALCAM-neutralizing antibodies prior to coculture. Representative flow histogram showing CFSE dilution (left) and proliferation index (right) of indicated groups of cultures. (**B**) CD4^+^CD25^−^ T cells were pre-treated with CD6-neutralizing antibodies or control isotype prior to coculture. Representative flow histogram showing CFSE dilution (left) and proliferation index (right) of indicated groups of cultures. Data from 3 independent experiments are shown. Data presented are mean ± SD. One-way ANOVA, *P < .05, **P < .01, ****P < .0001.

As T cells undergo clonal expansion following activation, we next investigated the effect of blocking ALCAM-CD6 pathway in modulating MSC function on CD4^+^ T-cell proliferation. To determine the effect of MSCs on T-cell proliferation, CFSE-labeled CD4^+^CD25^−^ T cells were stimulated with anti-CD3/28 for 72 h on an MSC monolayer. Our data showed that MSCs significantly suppressed CD4^+^ T-cell proliferation, compared to T cells stimulated alone (Fig. 3A). To assess the contribution of ALCAM-CD6 interaction in the observed MSC-mediated suppression of T-cell proliferation, MSC-T cell coculture assays were performed in the presence of ALCAM-neutralizing (Fig. 3A) and CD6-neutralizing antibodies (Fig. 3B) with their respective isotype controls. Anti-ALCAM antibody treatment resulted in the abrogation of MSC-mediated suppression of T-cell proliferation (Fig. 3A). Similarly, neutralization of CD6 resulted in a substantially higher T-cell proliferation index compared to control isotype treatment (Fig. 3B). Our data suggest that ALCAM-CD6 interaction is crucial for the suppressive function of MSCs on T-cell expansion.

### MSC-Expressed ALCAM is Indispensable for the Suppression of T-cell Activation and Expansion

**Figure 4.**
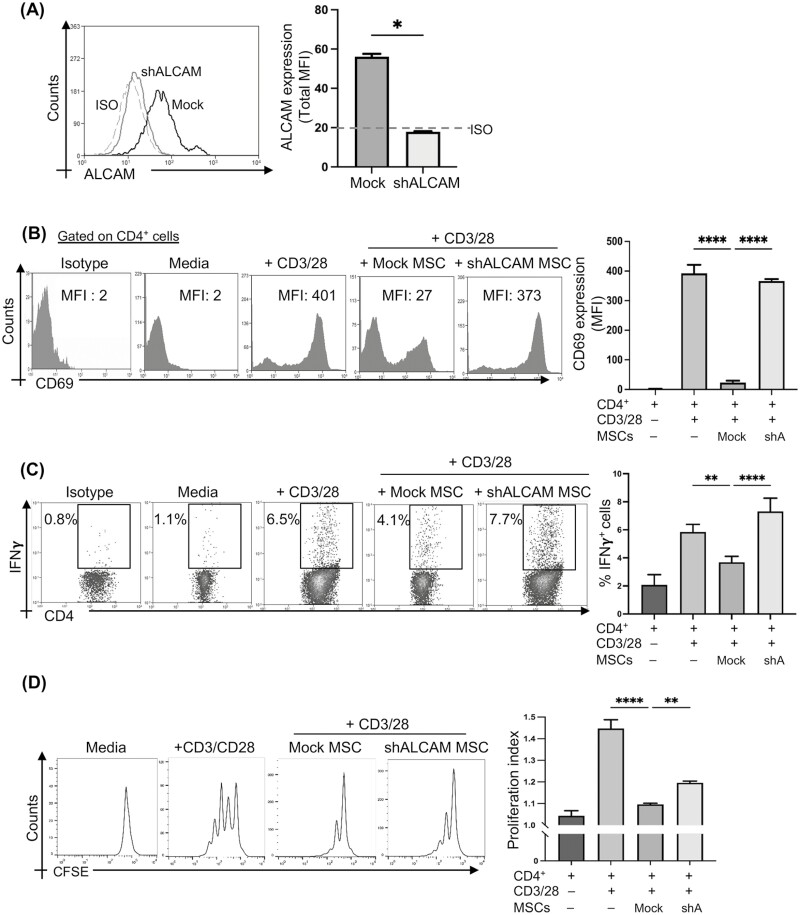
Silencing ALCAM expression on MSCs abrogates their capacity to suppress T-cell activation and proliferation. (**A**) Flow cytometry histogram (left) and bar graph (right) showing downregulation of ALCAM expression in mouse MSCs transfected with ALCAM-specific shRNA compared to control shRNA (Mock) on day 3 post-transfections to baseline levels (dotted line, isotype-stained control). Dotted line, isotype control; gray solid line, ALCAM-silenced MSCs, black solid line, Mock-silenced MSCs. CD4^+^CD25^−^ T cells were magnetically sorted from C57BL/6 splenocytes and stimulated with anti-CD3/CD28-coated beads at a 1:2 ratio and cultured on the monolayer of ALCAM- or Mock-silenced MSCs. (**B**) Representative flow cytometry histogram (left) and bar chart (right) demonstrating expression of CD69 (MFI) by CD4^+^ T cells in the indicated groups of cultures at 24 h. (**C**) Representative flow cytometry dot plots (left) and cumulative bar chart (right) showing frequencies of CD4^+^IFNγ^+^ Th1 cells (gated on CD4^+^ cells) in the indicated groups of cultures at 48 h of coculture. (**D**) CD4^+^CD25^−^ T cells were labeled with CellTrace CFSE prior to the coculture assays. Representative flow histogram showing CFSE dilution (left) and proliferation index (right) of indicated groups of cultures at 72 h. Data from 3 independent experiments are shown. Data presented are mean ± SD. One-way ANOVA, *P < .05, **P < .01, ****P < .0001.

Having confirmed the function of ALCAM in the human setting, we next sought to determine the alloimmune function of MSCs-expressed ALCAM in the in vivo settings. Thus, we silenced ALCAM expression in murine MSCs and characterized its function in our controlled murine MSC–T-cell coculture system. ALCAM shRNA transfection resulted in complete downregulation of ALCAM expression to baseline isotype-stained controls, compared to control shRNA treatment (Mock) (Fig. 4A). CD4^+^CD25^−^ T cells were purified from C57BL/6 spleens by magnetic sorting prior to co-culturing on an MSC monolayer. CD3/28-stimulated T cells were cocultured either with shALCAM or Mock MSCs for 24 h. CD4^+^ T cells cocultured with shALCAM MSCs showed comparable early T-cell activation with CD4^+^ T cells cultured without MSCs, as indicated by CD69 expression (Fig. 4B). However, T cells cocultured with Mock MSCs resulted in significant downregulation of CD69 expression (Fig. 4B). Moreover, MSC-mediated suppression of T-cell effector function was not observed in shALCAM MSC cocultures, as demonstrated by comparable frequencies of CD4^+^IFNγ^+^ T cells in shALCAM MSC cultures to CD3/28-stimulation only cultures (Fig. 4C). Similarly, ALCAM-silenced MSCs failed to suppress CD3/28-induced T-cell proliferation, compared to Mock-silenced MSCs which significantly suppressed T-cell expansion. Our genetically silenced MSCs experiment replicated the ALCAM-CD6 neutralization findings, suggesting surface expression of ALCAM is indispensable to the suppressive function of MSCs.

Silencing ALCAM expression on MSCs abrogates their capacity to suppress alloreactive T-cell generation and their infiltration to the site of alloantigen challenge.

**Figure 5.**
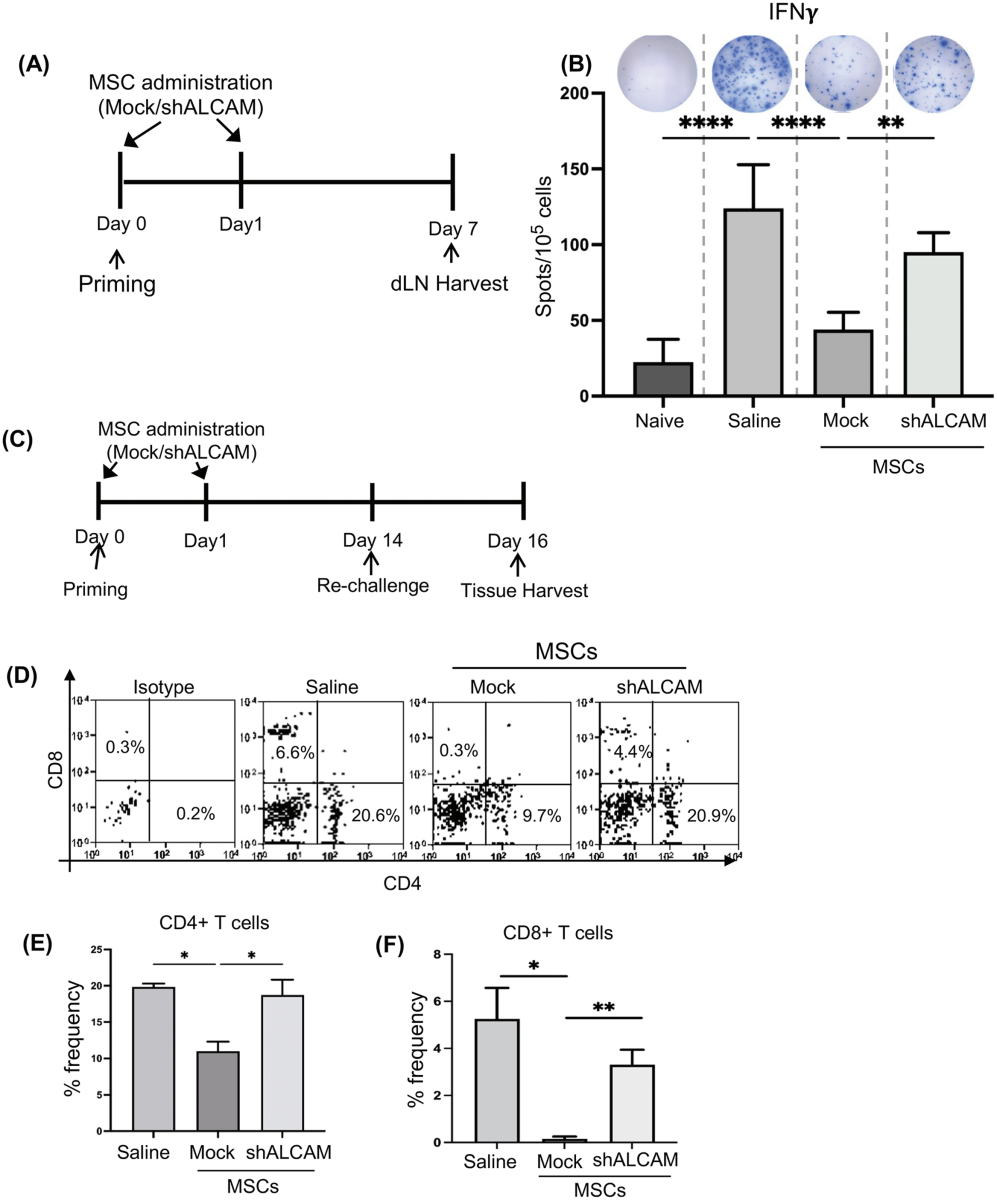
Lack of ALCAM expression reduces MSCs capacity to suppress generation and infiltration of alloreactive T cell. (**A**) Schematic of experimental design showing the time points of subcutaneous alloantigen (BALB/c splenocytes) and intravenous ALCAM- or Mock-silenced MSCs (C57BL/6) administration and harvesting of the draining lymph nodes (dLN) on day 7 post-sensitization. Saline treatment served as control. (**B**) Responder T cells were isolated from the draining lymph nodes of mice treated with ALCAM- or Mock-silenced MSCs 7 days after alloantigen sensitization and were stimulated with BALB/c splenic APCs. Representative well images (top) and bar graph quantifying the number of spots of responder cells per 100 000 T cells. (**C**) Experimental design showing the time points of intravenous ALCAM- or Mock-silenced MSCs administration, epicutaneous re-challenge and ear pinnae harvesting. Mice were primed via subcutaneous injection of alloantigen and re-challenged on day 14 post-sensitization and ear pinnae were harvested on day 16 post-sensitization. (**D**) Representative dot plots demonstrating frequencies of CD4^+^ T cells and CD8^+^ T cells in pinnae of indicated treatment groups. Cumulative bar chart showing frequencies of (**E**) CD4^+^ T cells and (**F**) CD8^+^ T cells in the re-challenged pinnae tissue harvested from indicated treatment groups of mice. The data shown are from 3 experiments, with each experiment consisting of 6 animals/group. Data presented are mean ± SEM. One-way ANOVA, *P < .05, **P < .01, ****P < .0001.

After observing ALCAM-silenced MSCs fail to suppress T-cell activation in vitro, we next investigated the role of ALCAM expression on MSCs in modulating alloimmunity in vivo by priming C57BL/6 mice with BALB/c alloantigens (splenocytes). As intravenously injected MSCs home to site of antigen presentation (Supplementary Fig. S3),^3,18^ we utilized IV injection to administer either ALCAM or control Mock-silenced MSCs at 1 and 24 h following priming (Fig. 5A). Single-cell suspensions of draining lymph nodes were prepared for ELISPOT analysis. To assess the specific effect on alloreactive T-cell generation, T cells were purified from the draining lymph nodes of the primed mice (C57BL/6) and cocultured in the presence of allogeneic stimulators (CD90.2^−^ splenocytes (APCs) from BALB/c mice) for 48 h. ELISPOT analysis showed an approximate 2.5-fold suppression in IFNγ secretion by the T cells isolated from Mock MSC-treated mice, compared to those isolated from saline-treated mice (Fig. 5B). However, this MSC-mediated suppression of alloreactive T-cell generation was absent in the T cells purified from ALCAM silenced-MSC treated mice (Fig. 5B). To further assess the effect of in vivo administration of MSCs on alloreactive T-cell generation and function, primed C57BL/6 mice treated with saline control, Mock or ALCAM-silenced MSCs, were re-challenged via epicutaneous ear injection of mitomycin-C treated BALB/c splenocytes on day 14 post-sensitization (Fig. 5C). Site of alloantigen challenge (right ear) was harvested and single-cell suspensions were prepared for flow cytometry analysis. Our data demonstrated that administration of Mock-MSCs resulted in a significant reduction in the frequencies of CD4^+^ T cell at the alloantigen challenge site, compared to the control saline-treated group (Fig. 5D, 5E).

Interestingly, silencing of ALCAM expression on MSCs abrogated the observed decreased frequencies of CD4^+^ T cells. Furthermore, Mock MSC treatment resulted in the complete suppression of CD8^+^ T-cell infiltration to the challenge site, compared to saline treatment (Fig. 5D, 5F). This suppressive effect of MSCs was not observed in CD8^+^ T cells purified from ALCAM-silenced MSC-treated mice (Fig. 5D, 5F). Taken together, our data suggest that surface expression of ALCAM is integral to MSC function in suppressing alloreactive T-cell generation and their infiltration into the site of alloantigen challenge.

### Genetic Silencing of ALCAM in MSCs Resulted in Loss of Their Capacity to Prevent Delayed Type-hypersensitivity Reaction

**Figure 6.**
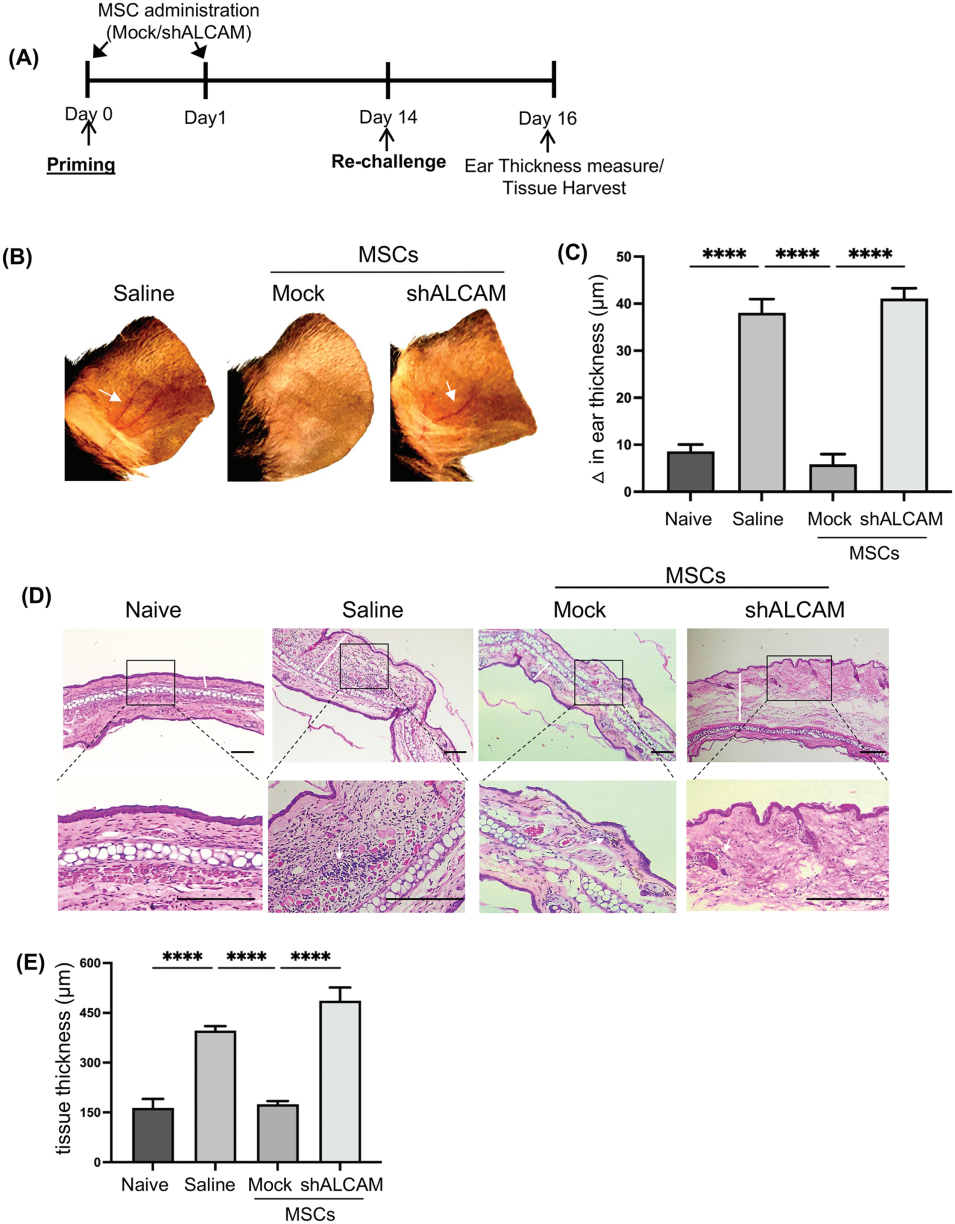
In vivo administration of ALCAM-silenced MSCs fail to prevent delayed-type hypersensitivity-mediated tissue inflammation and damage. (**A**) Experimental design showing the time points of intravenous ALCAM- or Mock-silenced MSCs administration. Mice were primed via subcutaneous injection of alloantigens and re-challenged on day 14 post-sensitization and ear thickness was measured and tissues were harvested on day 16 post-sensitization. (**B**) Representative pictures of re-challenged ear on day 16 post-sensitization showing inflamed and engorged vascularity (white arrows) in indicated treatment groups of mice. (**C**) Bar graph quantifying ear swelling response. Degree of swelling was calculated as the difference in thickness of the challenged ear (right ear) minus the baseline thickness of the contralateral control ear (left ear). (**D**) H&E pinnae cross-sections of different treatment groups to visualize tissue structure and infiltration of immune cells (scale bar = 100 μm). (**E**) Bar chart quantifying thickness of the epidermis and dermis. Thickness (as outlined by the white line) was measured from the epidermis to the cartilage using ImageJ software. The data shown is from 2 experiments, with each experiment consisting of 7-8 animals/group. Data presented are mean ± SEM. One-way ANOVA, ****P < .0001.

MSCs have been demonstrated to regulate alloimmunity by our group and others. Having observed that ALCAM-silenced MSCs fail to suppress alloreactive T-cell generation, we next assessed whether silencing ALCAM impairs the capacity of MSCs to prevent allosensitization using a well-established model of delayed-type hypersensitivity (DTH) response. Allosensitized C57BL/6 mice were re-challenged with mitomycin-C treated BALB/c splenocytes via epicutaneous injection in the right ear pinnae. Ear thickness was measured 48 h following the re-challenge (Fig. 6A). Alloantigen re-challenge resulted in significant inflammation as indicated by engorged vessels and area of rubor in the ears of saline- and ALCAM-silenced MSCs-treated mice; however, mice treated with Mock MSC showed minimal inflammation (Fig. 6B). Difference in ear thickness was calculated by subtracting the thickness in the challenged ear (right) with the saline-challenged contralateral ear (left). Mock MSC treatment significantly prevented tissue inflammation and edema, resulting in comparable ear thickness with the naïve mice (Fig. 6C). However, silencing the expression of ALCAM by MSCs significantly reduced their capacity to prevent DTH response, as shown by 4-fold higher difference in ear thickness, compared to Mock MSC treatment group (Fig. 6C).

To further visualize the extent of DTH-mediated tissue inflammation and damage, ears were harvested, and cross-sections were stained with H&E. Ears of mice treated with Mock MSCs showed comparable ear architecture to that of naïve mice without significant infiltration of immune cells (Fig. 6D). However, ears of mice treated with ALCAM-silenced MSCs showed significantly inflamed dermal layer marked with immune foci, similar to that of saline-treated mice (Fig. 6D). A significant 2.5-fold increase in dermal thickness was observed following re-challenge in saline-treated mice, which was absent in Mock-MSC treated mice (Fig. 6E). Mice treated with ALCAM-silenced MSCs, on the contrary, fail to suppress dermal inflammation (Fig. 6E). Our data demonstrate that MSC-mediated suppression of DTH response depends on the expression of ALCAM.

## Discussion

This study advances our understanding of the interaction between T cells and MSCs, in the setting of T-cell-mediated delayed type hypersensitivity. Our data demonstrate that MSCs directly interact with CD4^+^ T cells by binding to CD6 via their surface expression of ALCAM. Moreover, blockade of this interaction abrogates MSC-mediated suppression of T-cell function, as evidenced by reduced activation and expansion following neutralization of ALCAM or CD6 in MSC–T-cell cocultures. Furthermore, our in vivo data show that ALCAM is indispensable for MSCs’ capacity to prevent delayed-type hypersensitivty and suppress generation of alloreactive T cells and their tissue-damaging functions.

MSC-based cell therapy has garnered much attention in recent years for their immunomodulatory properties and capacity to migrate to inflamed tissues.^18,24,25^ Intravenously administered MSCs home to the site of alloantigen challenge and the draining lymphoid tissues following transplantation and DTH sensitization. After capturing and processing allograft antigens, mature APCs are trafficked to draining lymph nodes where they prime naïve host T cells, resulting in the generation of allogeneic IFNγ^+^ Th1 cells.^26^ As such, Th1 cells have been doted to be the primary cellular mediators of immune rejection.^27,28^ Recently, we and others have demonstrated that the administration of MSCs regulates adaptive immunity by suppressing APC activation and promoting regulatory T-cell function in a contact-dependent manner.^6,16^ Here, we demonstrate that MSCs regulate adaptive immunity by directly suppressing effector T-cell function in the setting of allogeneic delayed-type hypersensitivity response.

Recent reports have suggested the use of ALCAM as a phenotypic marker of human MSCs.^7^ Indeed, our observations show that hMSCs constitutively express ALCAM. Interestingly, our observations found murine MSCs similarly express high levels of ALCAM. Moreover, the current study is the first study to characterize the function of MSC-expressed ALCAM. Known to be expressed by various cell types, including leukocytes, dendritic cells, and endothelial cells, ALCAM binds to T-cell-expressed scavanger receptor CD6.^29^ Given the expression of its receptors CD6 on T cells and vascular endothelial cells, respectively, ALCAM has been implicated in T-cell function, leukocyte trafficking, and angiogenesis.^30,31^ Our data demonstrate that MSC-expressed ALCAM is critical to MSC–T-cell interaction, as demonstrated by the abrogation of MSC-mediated suppression of early T-cell activation following the blockade of ALCAM-CD6 interaction. Our finding along with the recent report showing loss of MSC-mediated suppression of CD4^+^ T-cell proliferation when MSCs were separated from CD4^+^ T cells using a Transwell,^32^ suggest the contact-dependent interaction of membrane-bound ALCAM with CD6 receptor on T cells is critical for MSC’s immunosuppressive capacity. Of note, the blockade of MSC-expressed ALCAM or its ligand CD6 on T cells equally resulted in the loss of MSC’s capacity to suppress the proliferation of CD4^+^ T cells and generation of IFNγ^+^-secreting CD4^+^ T cells. A previous study, however, has reported that ALCAM on dendritic cells interacts with T-cell-expressed CD6 to promote T-cell-mediated inflammation.^33^ Due to conflicting findings, the immunoregulatory function of CD6 has been a topic of much debate. Indeed, one study has demonstrated the inhibitory function of CD6 on early T-cell activation,^11^ while another study has shown CD6 provides co-stimulatory signals to enhance T-cell activation.^34^ Interestingly, a recent landmark study utilizing CD6-deficient CD4^+^ T cells demonstrated that CD6 deficiency on CD4^+^ T cells enhances T-cell activation,^10^ implicating CD6 as a negative regulator of CD4^+^ T-cell activation. Our findings corroborate this study, highlighting the significant contribution of ALCAM-CD6 interaction in the context of MSC-mediated immunoregulation of T cells.

To translate our in vitro findings into in vivo settings, we used an allosensitization model of DTH, in which pathogenesis is characterized by massive infiltration of alloreactive CD4^+^ and CD8^+^ T cells targeting the site of alloantigen challenge, resulting in significant ear swelling and inflammation in the draining lymph nodes. Murine model of delayed-type hypersensitivity allows for clinical and cellular assessment of antigen-specific CD4^+^ and CD8^+^ T-cell-mediated immune response.^22,35^ MSCs were silenced for ALCAM expression with short hairpin RNA and administered to allosensitized mice shortly after alloantigen priming. In accordance with our antibody-mediated neutralization in our controlled human cell settings, murine in vivo data demonstrated that the knockdown of ALCAM on MSCs abrogates the capacity of MSCs to suppress the infiltration of alloreactive T cells. Detailed analysis of T-cell subsets showed that Mock-MSCs inhibit the infiltration of CD4^+^ and CD8^+^ T cells into the site of the alloantigen challenge, which was not observed following ALCAM-silenced MSC treatment. In the setting of alloimmmune response, Th1 cells play a pivotal role in alloantigen-induced inflammatory tissue damage by secretion of IFNγ and generation of CD8-mediated immune responses.^36,37^ Our findings showing significantly higher levels of IFNγ-secreting T cells in the draining lymph nodes of mice treated with ALCAM-silenced MSCs compared to Mock-silenced MSCs suggest ALCAM expression is critical for MSC-mediated suppression of IFNγ secretion by-alloreactive T cells including CD4^+^ and CD8^+^ cells.

Furthermore, our experiments evaluating the effect of MSC-expressed ALCAM on alloreactive T-cell-mediated tissue swelling and damage to the site of alloantigen challenge showed complete abrogation of tissue edema and engorged blood vessel of the challenged ear in the mice treated Mock-silenced MSCs. Consistent with our observation of reduced infiltration of T cells, the tissue-protective effects of MSCs were not observed in mice treated with ALCAM-silenced MSCs. Indeed, histopathology analysis showed a marked reduction in epidermal damage and dermal thickness accompanied by minimal cellular infiltrates in the alloantigen-challenged tissues of the Mock-silenced MSCs group, an effect not observed in the ALCAM-silenced MSCs group. Our findings not only corroborate our flow cytometry analysis of T-cell infiltration but also suggest a substantial contribution of MSC-expressed ALCAM on regulating alloreactive T-cell-mediated tissue inflammation and damage.

MSCs exert their immunoregulatory activity via a diverse array of mechanisms involving both the secretion of soluble factors and surface expression of immunomodulatory ligands.^2^ Our study demonstrates the undefined function of ALCAM expression on MSCs and further demonstrates that MSCs directly regulate the activation and expansion of Th1 cells through the ALCAM-CD6 pathway. Furthermore, our data establish that the capacity of MSCs to suppress the generation of alloreactive Th1 cells and T-cell-mediated delayed-type hypersensitivity response depends on their surface expression of ALCAM, providing novel insights into the development of MSC-based therapy for regulating alloimmunity.

## Supplementary Material

szad012_suppl_Supplementary_Material

## Data Availability

The data that support the findings of this study are available in the methods of this article. Detailed data that support the findings of this study are available from the corresponding author upon reasonable request.

## References

[1] Salem HK , Thiemermann C. Mesenchymal stromal cells: current understanding and clinical status. Stem Cells. 2009;28(3):585-596. 10.1002/stem.269 PMC2962904 19967788

[2] Nauta AJ , Fibbe WE. Immunomodulatory properties of mesenchymal stromal cells. Blood. 2007;110(10):3499-3506. 10.1182/blood-2007-02-069716 17664353

[3] Omoto M , Katikireddy KR, Rezazadeh A, Dohlman TH, Chauhan SK. Mesenchymal stem cells home to inflamed ocular surface and suppress allosensitization in corneal transplantation. Invest Ophthalmol Vis Sci. 2014;55(10):6631-6638. 10.1167/iovs.14-15413 25228546

[4] Amouzegar A , Mittal SK, Sahu A, Sahu SK, Chauhan SK. Mesenchymal stem cells modulate differentiation of myeloid progenitor cells during inflammation. Stem Cells. 2017;35(6):1532-1541. 10.1002/stem.2611 28295880 PMC5446294

[5] Jiang D , Muschhammer J, Qi Y, et al. Suppression of neutrophil-mediated tissue damage-a novel skill of mesenchymal stem cells. Stem Cells. 2016;34(9):2393-2406. 10.1002/stem.2417 27299700 PMC5572139

[6] Mittal SK , Cho WK, Elbasiony E, et al. Mesenchymal stem cells augment regulatory T cell function via CD80-mediated interactions and promote allograft survival. Am J Transplant. 2022;22(6):1564-1577. 10.1111/ajt.17001 35170213 PMC11261724

[7] Brinkhof B , Zhang B, Cui Z, Ye H, Wang H. ALCAM (CD166) as a gene expression marker for human mesenchymal stromal cell characterisation. Gene X. 2020;5:100031. 10.1016/j.gene.2020.100031 32550557 PMC7285916

[8] Bowen MA , Patel DD, Li X, et al. Cloning, mapping, and characterization of activated leukocyte-cell adhesion molecule (ALCAM), a CD6 ligand. J Exp Med. 1995;181(6):2213-2220. 10.1084/jem.181.6.2213 7760007 PMC2192054

[9] Chappell PE , Garner LI, Yan J, et al. Structures of CD6 and its ligand CD166 give insight into their interaction. Structure. 2015;23(8):1426-1436. 10.1016/j.str.2015.05.019 26146185 PMC4533223

[10] Li Y , Singer NG, Whitbred J, et al. CD6 as a potential target for treating multiple sclerosis. Proc Natl Acad Sci USA. 2017;114(10):2687-2692. 10.1073/pnas.1615253114 28209777 PMC5347585

[11] Oliveira MI , Gonçalves CM, Pinto M, et al. CD6 attenuates early and late signaling events, setting thresholds for T-cell activation. Eur J Immunol. 2012;42(1):195-205. 10.1002/eji.201040528 21956609 PMC3298641

[12] Singer NG , Richardson BC, Powers D, et al. Role of the CD6 glycoprotein in antigen-specific and autoreactive responses of cloned human T lymphocytes. Immunology. 1996;88(4):537-543. 8881754 PMC1456636

[13] Bott CM , Doshi JB, Morimoto C, Romain PL, Fox DA. Activation of human T cells through CD6: functional effects of a novel anti-CD6 monoclonal antibody and definition of four epitopes of the CD6 glycoprotein. Int Immunol. 1993;5(7):783-792. 10.1093/intimm/5.7.783 7690243

[14] Reis M , Mavin E, Nicholson L, et al. Mesenchymal stromal cell-derived extracellular vesicles attenuate dendritic cell maturation and function. Front Immunol. 2018;9:2538. 10.3389/fimmu.2018.02538 30473695 PMC6237916

[15] Skelsey ME , Mellon J, Niederkorn JY. Gamma delta T cells are needed for ocular immune privilege and corneal graft survival. J Immunol. 2001;166(7):4327-4333. 10.4049/jimmunol.166.7.4327 11254685

[16] Mittal SK , Foulsham W, Shukla S, et al. Mesenchymal stromal cells modulate corneal alloimmunity via secretion of hepatocyte growth factor. Stem Cells Transl Med. 2019;8(10):1030-1040. 10.1002/sctm.19-0004 31179638 PMC6766689

[17] Dominici M , le Blanc K, Mueller I, et al. Minimal criteria for defining multipotent mesenchymal stromal cells. The International Society for Cellular Therapy position statement. Cytotherapy. 2006;8(4):315-317. 10.1080/14653240600855905 16923606

[18] Shukla S , Mittal SK, Foulsham W, et al. Therapeutic efficacy of different routes of mesenchymal stem cell administration in corneal injury. Ocul Surf. 2019;17(4):729-736. 10.1016/j.jtos.2019.07.005 31279065 PMC6874749

[19] Broggi A , Cigni C, Zanoni I, et al. Preparation of single-cell suspensions for cytofluorimetric analysis from different mouse skin regions. J Vis Exp. 2016;(110):e52589. 27166881 10.3791/52589 PMC4941935

[20] Cho WK , Mittal SK, Elbasiony E, et al. Ocular surface mast cells promote inflammatory lymphangiogenesis. Microvasc Res. 2022;141:104320. 10.1016/j.mvr.2022.104320 35031275 PMC8923954

[21] Cho WK , Mittal SK, Elbasiony E, Chauhan SK. Activation of ocular surface mast cells promotes corneal neovascularization. Ocul Surf. 2020;18(4):857-864. 10.1016/j.jtos.2020.09.002 32916251 PMC7686271

[22] Li M , Mittal SK, Foulsham W, et al. Mast cells contribute to the induction of ocular mucosal alloimmunity. Am J Transplant. 2019;19(3):662-673. 30129280 10.1111/ajt.15084 PMC7941346

[23] Shukla S , Cho WK, Elbasiony E, et al. Non-immune and immune functions of interleukin-36γ suppress epithelial repair at the ocular surface. FASEB J. 2022;36(8). 10.1096/fj.202200174RR PMC9924024 35781326

[24] Gao F , Chiu SM, Motan DAL, et al. Mesenchymal stem cells and immunomodulation: current status and future prospects. Cell Death Dis. 2016;7(1):e2062. 10.1038/cddis.2015.327 26794657 PMC4816164

[25] Nitzsche F , Müller C, Lukomska B, et al. Concise review: MSC adhesion cascade-insights into homing and transendothelial migration. Stem Cells. 2017;35(6):1446-1460. 10.1002/stem.2614 28316123

[26] Amouzegar A , Chauhan SK, Dana R. Alloimmunity and tolerance in corneal transplantation. J Immunol. 2016;196(10):3983-3991. 10.4049/jimmunol.1600251 27183635 PMC4874505

[27] Nickerson P , Steurer W, Steiger J, et al. Cytokines and the Th1/Th2 paradigm in transplantation. Curr Opin Immunol. 1994;6(5):757-764. 10.1016/0952-7915(94)90081-7 7826531

[28] Illigens BM , Yamada A, Anosova N, et al. Dual effects of the alloresponse by Th1 and Th2 cells on acute and chronic rejection of allotransplants. Eur J Immunol. 2009;39(11):3000-3009. 10.1002/eji.200838980 19658090 PMC2911804

[29] Ferragut F , Vachetta VS, Troncoso MF, Rabinovich GA, Elola MT. ALCAM/CD166: A pleiotropic mediator of cell adhesion, stemness and cancer progression. Cytokine Growth Factor Rev. 2021;61:27-37. 10.1016/j.cytogfr.2021.07.001 34272152

[30] Ohneda O , Ohneda K, Arai F, et al. ALCAM (CD166): its role in hematopoietic and endothelial development. Blood. 2001;98(7):2134-2142. 10.1182/blood.v98.7.2134 11568000

[31] Cayrol R , Wosik K, Berard JL, et al. Activated leukocyte cell adhesion molecule promotes leukocyte trafficking into the central nervous system. Nat Immunol. 2008;9(2):137-145. 10.1038/ni1551 18157132

[32] Lynch K , Treacy O, Chen X, et al. TGF-β1-licensed murine MSCs show superior therapeutic efficacy in modulating corneal allograft immune rejection in vivo. Mol Ther. 2020;28(9):2023-2043. 10.1016/j.ymthe.2020.05.023 32531237 PMC7474271

[33] Zimmerman AW , Joosten B, Torensma R, et al. Long-term engagement of CD6 and ALCAM is essential for T-cell proliferation induced by dendritic cells. Blood. 2006;107(8):3212-3220. 10.1182/blood-2005-09-3881 16352806

[34] Orta-Mascaró M , Consuegra-Fernández M, Carreras E, et al. CD6 modulates thymocyte selection and peripheral T cell homeostasis. J Exp Med. 2016;213(8):1387-1397. 10.1084/jem.20151785 27377588 PMC4986531

[35] Akahira-Azuma M , Szczepanik M, Tsuji RF, et al. Early delayed-type hypersensitivity eosinophil infiltrates depend on T helper 2 cytokines and interferon-γ via CXCR3 chemokines. Immunology. 2004;111(3):306-317. 10.1111/j.0019-2805.2004.01818.x 15009431 PMC1782430

[36] Smeltz RB , Chen J, Ehrhardt R, Shevach EM. Role of IFN-γ in Th1 differentiation: IFN-γ regulates IL-18Rα expression by preventing the negative effects of IL-4 and by inducing/maintaining IL-12 receptor β2 expression. J. Immun. J. 2002;168(12):6165-6172. 10.4049/jimmunol.168.12.6165 12055229

[37] Janeway CA Jr , Travers P, Walport M, et al. Immunobiology: the immune system in health and disease. 5th edition. New York: Garland Science; 2001. Macrophage activation by armed CD4 TH1 cells. https://www.ncbi.nlm.nih.gov/books/NBK27153/

