# Supplementary material for: Mesenchymal Stromal Cells Suppress T-Cell-Mediated Delayed-Type Hypersensitivity via ALCAM-CD6 Interaction
Source: Stem Cells Transl Med. 2023 Apr 17;12(4):221–33. doi: 10.1093/stcltm/szad012 (PMC10108723; doi:10.1093/stcltm/szad012)
Supplement: szad012_suppl_Supplementary_Material [file szad012_suppl_supplementary_material.pdf]

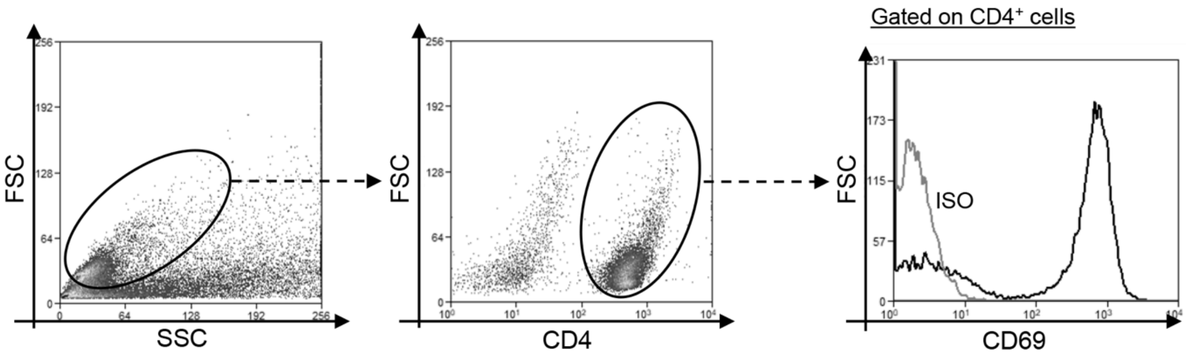

Supplementary Figure S1. Gating strategies for CD4$^+$CD69$^+$ T cells

Representative dot plots demonstrating the gating strategy for the characterization of CD4$^+$CD69$^+$ T cells in single cell suspensions.

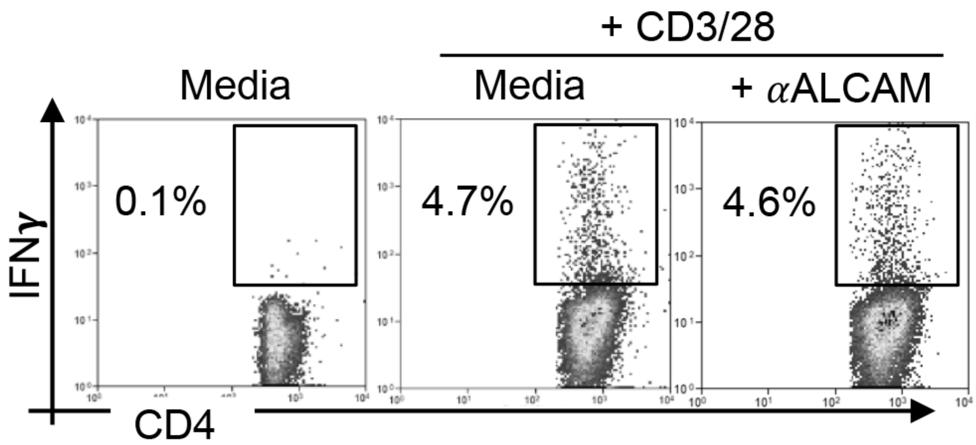

Supplementary Figure S2. ALCAM-neutralizing antibody has no effect on CD3/28-mediated activation of $CD4^+$ T cell function

$CD4^+CD25^-$ T cells were magnetically sorted from human peripheral blood mononuclear cells and stimulated with anti-CD3/CD28-coated beads at a 1:1 ratio. $CD4^+$ T cells were cultured with or without ALCAM-neutralizing antibodies. Representative flow cytometry dot plots showing frequencies of $CD4^+IFN\gamma^+$ Th1 cells (gated on $CD4^+$ cells) in the indicated groups of cultures at 48 hours of co-culture.

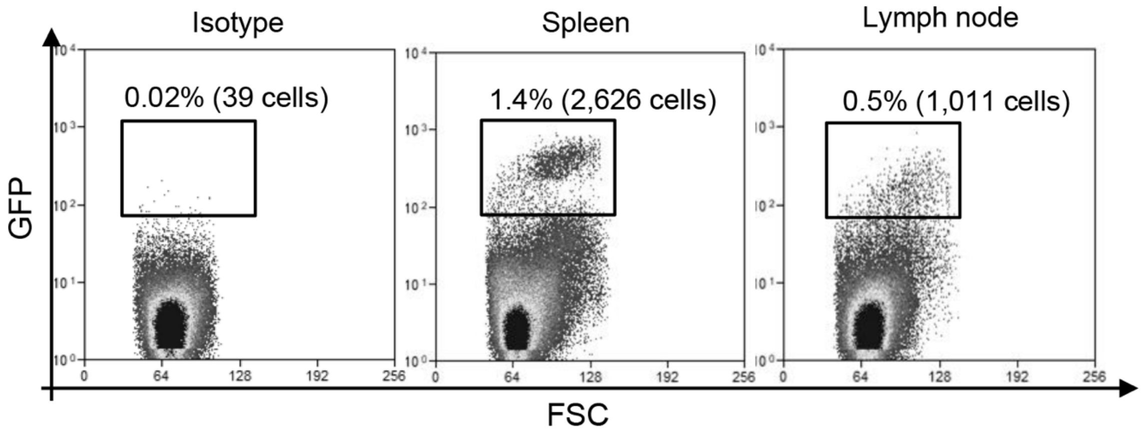

Supplementary Figure S3. MSCs home to draining lymphoid tissues

GFP-tagged MSCs ($0.5x10^6$ cells) were injected intravenously following alloantigen presentation. Lymphoid tissues including the spleen and draining cervical lymph nodes were harvested three days following MSC treatment. Single cell suspensions were prepared and number of GFP^+ cells were quantified using flow cytometry.
